# Enhanced catalytic degradation of amoxicillin with TiO_2_–Fe_3_O_4_ composites *via* a submerged magnetic separation membrane photocatalytic reactor (SMSMPR)

**DOI:** 10.1039/c9ra00158a

**Published:** 2019-04-23

**Authors:** Qilong Li, Hui Kong, Rongrong Jia, Jiahui Shao, Yiliang He

**Affiliations:** School of Environmental Science and Engineering, Shanghai Jiao Tong University 800 Dongchuan Rd Shanghai 200240 China ylhe@sjtu.edu.cn +86-021-54744008; School of Naval Architecture, Ocean and Civil Engineering, Shanghai Jiao Tong University Shanghai 200030 PR China

## Abstract

A novel photo-Fenton catalytic system for the removal of organic pollutants was presented, including the use of photo-Fenton process and a submerged magnetic separation membrane photocatalytic reactor (SMSMPR). We synthesized TiO_2_–Fe_3_O_4_ composites as the photocatalyst and made full use of the magnetism of the photocatalyst to realize the recollection of the catalyst from the medium, which is critical to the commercialization of photocatalytic technology for wastewater treatment. The photo-Fenton performance of TiO_2_–Fe_3_O_4_ is evaluated with amoxicillin trihydrate (AMX) as a target pollutant. The results indicate that the TiO_2_–Fe_3_O_4_/H_2_O_2_ oxidation system shows efficient degradation of AMX. Fe_3_O_4_ could not only enhance the heterogeneous Fenton degradation of organic compounds but also allow the photocatalyst to be magnetically separated from treated water. After four reaction cycles, the TiO_2_–Fe_3_O_4_ composites still exhibit 85.2% removal efficiency of AMX and show excellent recovery properties. Accordingly, the SMSMPR with the TiO_2_–Fe_3_O_4_ composite is a promising way for removing organic pollutants.

## Abstract 2

With a TiO_2_–Fe_3_O_4_ composite as the catalyst, amoxicillin was degraded *via* a photo-Fenton process using a submerged magnetic separation membrane photocatalytic reactor.

## Graphical abstract

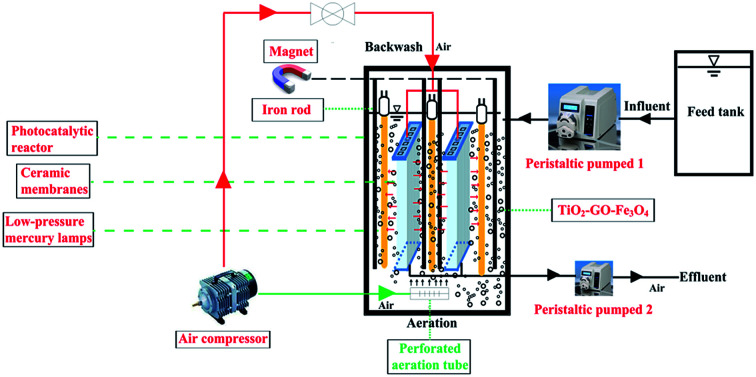

## 1. Introduction

The penetration of pharmaceuticals into the environment has drawn considerable attention in recent years.^1–3^ Several effective alternative water treatment technologies have been considered in recent studies, including adsorption,^4,5^ membrane filtration,^6–8^ coagulation^9^ and advanced oxidation technologies.^10–12^ Photocatalytic environmental remediation has always been the research focus since the breakthrough of water splitting was covered by Fujishima and Honda in 1972.^13,14^ Various photocatalysts have been used in the photocatalysis process, among which titanium dioxide (TiO_2_) is the most widely studied and applied owing to its advantages of strong oxidizing ability, chemical stability, nontoxicity, low cost and high hydrophilicity.^13,15^ The photo-Fenton process is a typical combination of two kinds of advanced oxidation process (AOP) and shows high oxidative removal efficiency of organics because of the highly enhanced generation of reactive hydroxyl radicals (·OH).^16–18^ However, one of the major drawbacks, separation and recycling of catalyst particles from large quantities of water, needs further cost and restrains the practical application of photocatalytic process. Accordingly, many studies were trying to explore an economic way to get the catalysts back. The ternary magnetic composite of Fe_3_O_4_@TiO_2_/SiO_2_ was prepared and used to remove Rhodamine B from wastewater. Results indicated that the Fe_3_O_4_@TiO_2_/SiO_2_ showed high photocatalytic activity and most importantly, it was recyclable.^19^ Fan *et al.* coated Fe_3_O_4_/SiO_2_ magnetic core with titania using hydrothermal synthetic method. With external magnetic field, the Fe_3_O_4_/SiO_2_/TiO_2_ magnetic nanocomposites could be separated from the suspension successfully. TiO_2_ decorated with Fe_3_O_4_ could generate greater photocatalytic activity as Fe^3+^ can occupy both of the electron capture position and hole capture position, resulting in the decrease of electron–hole pair recombination of TiO_2_. However, aggregation of TiO_2_ particles can influence the optical properties and photoactivity of catalyst,^20^ meaning the necessary of preventing the particles from aggregation during the photocatalysis process.

Herein, we developed a special combination of TiO_2_–Fe_3_O_4_ catalysts, photo-Fenton process and SMSMPR. The incorporation of Fe_3_O_4_ can improve the catalytic activity of TiO_2_ (based on the energy level theory), and endowed TiO_2_ with excellent reusability with the help of external magnetic field.^19^ The two associated oxidation process, photocatalysis and Fenton oxidation can ensure the degradation of organics in an efficient way. The well-designed submerged magnetic separation membrane photocatalytic reactor can realize the waste water treatment and the separation of catalyst. The aeration in the reactor during the photocatalysis process will alleviate the agglomeration of TiO_2_ particles, which is essential to guarantee the specific surface area of photocatalyst. Also, the built-in backwashing treatment inside the SMSMPR could enhance the self-purification ability of membranes. We also evaluated the main factors influencing the removal of organics, tried to find out the optimum reaction condition and explored the possibility of cyclic utilization of the reaction system.

## 2. Materials and methods

### 2.1 Chemicals

Ferric nitrate (Fe(NO_3_)_3_·9H_2_O) and ethylene glycol (EG) were purchased from Shanghai Macklin Biochemical Co., Ltd. TiO_2_ was obtained from Degussa AG, Germany. Amoxicillin trihydrate (99.5%, k AMX), hydrogen peroxide (30%) and ethanol were supplied by Sinopharm Chemical Reagent Co., Ltd (Shanghai, China).

### 2.2 Synthesis of TiO_2_–Fe_3_O_4_

TiO_2_–Fe_3_O_4_ particles were synthesized by a facile hydrothermal synthetic method.^21^ Firstly, 200 mL mixture of ethanol and water (v:v = 1 : 1) was prepared. And different amounts of TiO_2_ and 5 mM Fe(NO_3_)_3_·9H_2_O were distributed in above solution by ultrasonic treatment for 3 h. Then it was dried at 60 °C, ground to powder and placed into a beaker, which was put in a 500 mL Teflon-lined autoclave. 60 mL ammonia solution was added into the autoclave beforehand and then sealed after the beaker was introduced. A drying oven was used to maintain a constant temperature for alkaline treatment in autoclave (180 °C for 12 h). Afterwards, the obtained samples were annealed at 200 °C for 5 h and the obtained powder was distributed in 100 mL EG by ultrasonic treatment for 3 h, after which the mixture was transferred to a 500 mL Teflon-lined autoclave and maintained at 180 °C for another 12 h. Finally, the obtained precipitates were washed three times with ethanol and distilled water and dried at 60 °C in vacuum. The final as-prepared products contained 10, 15, 20 and 25 wt% Fe_3_O_4_, respectively.

### 2.3 Characterization

Scanning electronic microscope (SEM, JSM 7800F), transmission electron microscope (TEM) and high resolution transmission electron microscope (HRTEM, JEM-2100F) images were obtained to characterize the morphological features of samples. X-ray diffraction (XRD) was carried out using a Bruker D8 Advance X-ray diffractometer with Cu-Kα radiation over a scan rate of 10° min^−1^ in the 2*θ* range from 5° to 80°. A Tensor 27 Fourier-transform infrared spectroscopy (FTIR) spectrometer (Nicolet 6700) was used to record the FTIR spectra of catalyst. X-ray photoelectron spectroscopy (XPS) analysis (PerkinElmer PHI 5000C, AlKα) of TiO_2_–Fe_3_O_4_ nanocomposites was also performed.

### 2.4 Photo-Fenton performance using SMSMPR

**Fig. 1:**
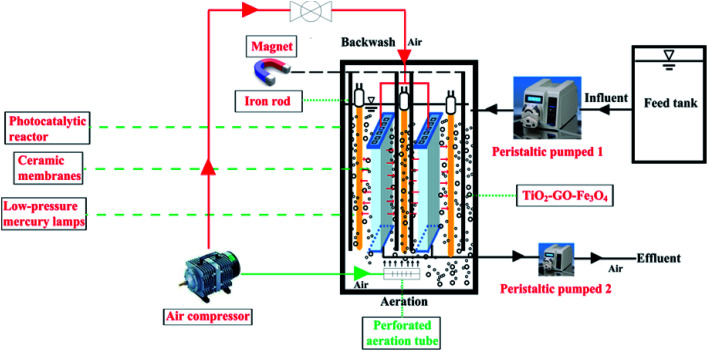
Schematic illustration of the submerged magnetic separation membrane photocatalytic reactor (SMSMPR).

The photo-Fenton process was performed with the SMSMPR and a schematic illustration of the SMSMPR for organic contaminant removal was depicted in Fig. 1. In this study, AMX (30 mg L^−1^) was chosen as the model organic contaminant. The concentration of AMX was measured by the total organic carbon (TOC) using a total organic carbon analyzer (multi 3100, Analytik Jena, Germany). Firstly, a peristaltic pump was utilized to pump AMX from the feed tank into an stainless steel container (*L* × *W* × *H* = 200 mm × 200 mm × 500 mm) at a flow rate of 100 mL min^−1^, which accommodated low-pressure mercury lamps (TUV, Philips), perforated aeration pipes, the hollow ceramic membranes (150 mm in length, 120 mm in width, and 4 mm in thickness), a powerful magnet connected to an iron rod and the mixture of organic solution and catalyst. Low-pressure mercury lamps (LPML; Philips) were placed around the ceramic membrane for photocatalysis and the light intensity quantitatively of a 100 W LPML was 1200 mW cm^−2^. The perforated aeration pipes, connected to an air compressor, was placed at the bottom of the reactor to produce dissolved oxygen-rich micro-porous bubbles, which can keep the catalyst from gathering and settling down to the bottom. After the wastewater was successfully pumped into the reactor, the catalyst was dosed in and the air compressor was opened to fluidize the catalysts. The low-pressure mercury lamps were switched on and the photocatalysis began after 45 min, during which time the pharmaceutical solution and catalyst can be well-mixed and reach the equilibrium. As for the ceramic membranes, they were used for the separation of effluents from the catalyst slurry. After the photo-Fenton process finished, another peristaltic pump was used to pump the effluent out. Furthermore, the ceramic membranes were connected to the air compressor and can be used for backwashing, making the cyclic utilization of the system possible. Finally, an external magnetic field, provided by a powerful magnet connected to an iron rod was also employed to realize the recollection of catalysts.

## 3. Results and discussion

**Fig. 2:**
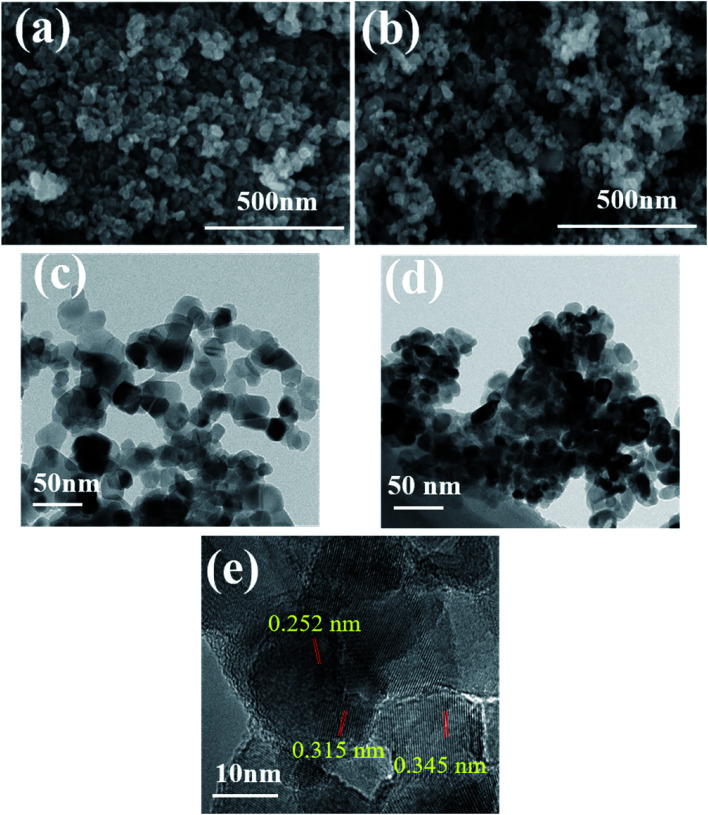
SEM and TEM images of pure TiO_2_ (a and c) and TiO_2_/15 wt% Fe_3_O_4_ (b and d) and (e) HRTEM images of TiO_2_/15 wt% Fe_3_O_4_.

Microimages of chemicals can provide its visual and intuitive morphology. The morphologies of photocatalysts were evaluated by SEM and TEM, and the corresponding images are presented in Fig. 2. Based on Fig. 2a and c, pure TiO_2_ had irregularly spherical shape with average diameter of 40 nm and the TiO_2_ particles were uniformly distributed in the plane. After combined with Fe_3_O_4_ (Fig. 2b and d), the nanoparticles showed obvious aggregation effect and smaller dimension, representing the presence of Fe_3_O_4_ in the composites and interaction between TiO_2_ and Fe_3_O_4_. Actually, during the formation of TiO_2_/Fe_3_O_4_ composites, the positively charged Fe^3+^ and negatively charged TiO_2_ can be attracted by each other and connected in the beginning. As the temperature becoming higher, the evaporated ammonia reacted with Fe^3+^ to produce Fe(OH)_3_ on the surface of TiO_2_ and the Fe(OH)_3_ were *in situ* formed on TiO_2_. After the annealed treatment at 200 °C for 5 h, the obtained Fe(OH)_3_ was decomposed to Fe_2_O_3_. Afterwards, EG was used as a reductant and effectively reduce Fe_2_O_3_ to Fe_3_O_4_ nanoparticles during the further hydrothermal process. Then TiO_2_–Fe_3_O_4_ composite was obtained. Accordingly, the driving force for the formation of TiO_2_–Fe_3_O_4_ composite was attributed to the attraction between the positively charged Fe^3+^ on Fe_3_O_4_ and negatively charged defects on the TiO_2_ surface. Fig. 2e revealed the interplanar distance of 0.345 nm and 0.315 nm, corresponding to the anatase (101) and rutile (101) planes of TiO_2_, respectively. As for the lattice fringes of 0.252 nm, they were characteristic of (311) crystal planes of cubic Fe_3_O_4_.^22,23^

**Fig. 3:**
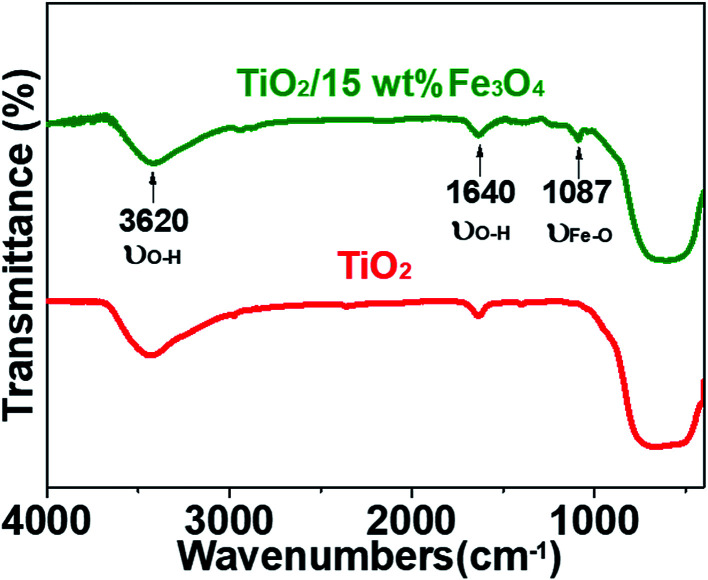
FTIR spectra of pure TiO_2_ and TiO_2_/15 wt% Fe_3_O_4_ composites.

**Fig. 4:**
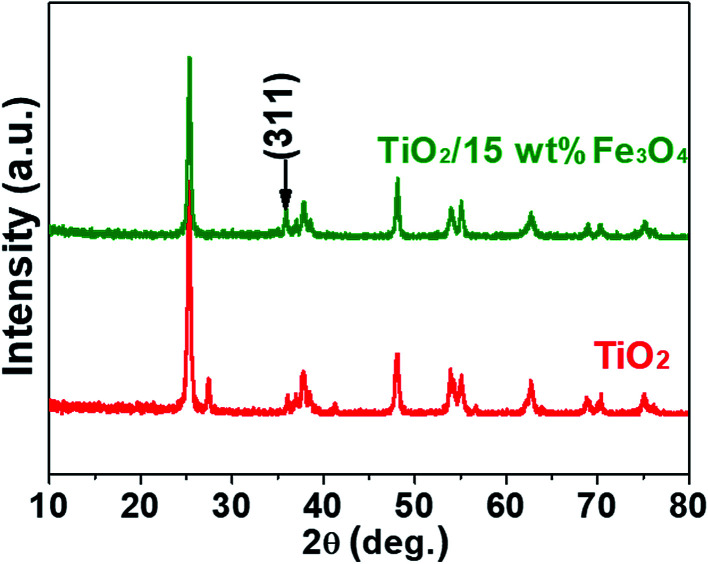
XRD patterns of pure TiO_2_ and TiO_2_/15 wt% Fe_3_O_4_ composites.

Fig. 3 and 4 depict the FTIR spectra and XRD patterns of pure TiO_2_ and TiO_2_/15 wt% Fe_3_O_4_, respectively. The obviously observed absorption band at 1640 and 3420 cm^−1^ in both of the catalysts corresponded to the bending vibration and symmetric stretching vibration of OH and surface absorbed water.^19,24,25^ Compared with FTIR spectra of pure TiO_2_, the peaks displayed in the FTIR spectra of TiO_2_/15 wt% Fe_3_O_4_ composites arose from not only OH group but also Fe–O vibration at 1087 cm^−1^.^26^ As displayed in Fig. 4, the peaks located at 2*θ* values of about 25.4°, 27.5°, 36.2°, 37.8°, 48.2° and 54.4° are the (101), (110), (101), (004), (200) and (204) diffraction peaks of TiO_2_. The intense and sharp peaks implied that the structure of TiO_2_ was well crystallized. The addition of Fe_3_O_4_ lowered the peak intensity of TiO_2_. In some other studies in the literature, the Fe_3_O_4_ characteristic peak of crystalline phase containing Fe_3_O_4_ could not be observed due to the low Fe_3_O_4_ content.^21,27^ In this paper, Similar result was found, the XRD characteristic diffraction peaks of Fe_3_O_4_ in TiO_2_/15 wt% Fe_3_O_4_ composites being not obvious because of the low loading content and high dispersion of Fe_3_O_4_. However, (311) crystal planes of Fe_3_O_4_ at 2*θ* = 35.86°,^22^ was slightly higher than that of pure TiO_2_, indicating the combination of TiO_2_ and Fe_3_O_4_.

**Fig. 5:**
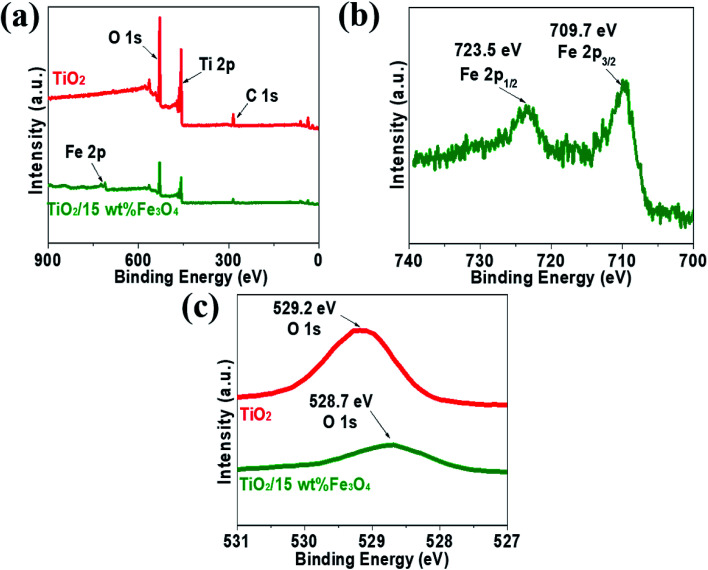
The XPS spectrum of pure TiO_2_ and TiO_2_/15 wt% Fe_3_O_4_ composites (a) Fe 2p from TiO_2_/15 wt% Fe_3_O_4_ composites (b) and O 1s from pure TiO_2_ and TiO_2_/15 wt% Fe_3_O_4_ composites (c).

XPS measurements were carried out to analyse the compositional and chemical states of samples and the results are shown in Fig. 5. Fig. 5a presents XPS spectrum of pure TiO_2_ and TiO_2_/15 wt% Fe_3_O_4_ composites. The full-scale XPS spectra indicated that there were elements O, Ti and C in TiO_2_, while TiO_2_/15 wt% Fe_3_O_4_ composites showed additional peaks of Fe 2p. The peak positions of Fe 2p_3/2_ and Fe 2p_1/2_ were 709.7 eV and 723.5 eV, respectively, which coincided with the previous literature.^28^ Actually, Fe_3_O_4_ should be described as FeO·Fe_2_O_3_, so the peaks are the comprehensive performance of Fe^2+^ and Fe^3+^. As for O 1s (Fig. 5c), the peak of pure TiO_2_ shifted from 529.2 eV to 528.7 eV for TiO_2_/15 wt% Fe_3_O_4_, implying the interaction between TiO_2_ and Fe_3_O_4_.

**Fig. 6:**
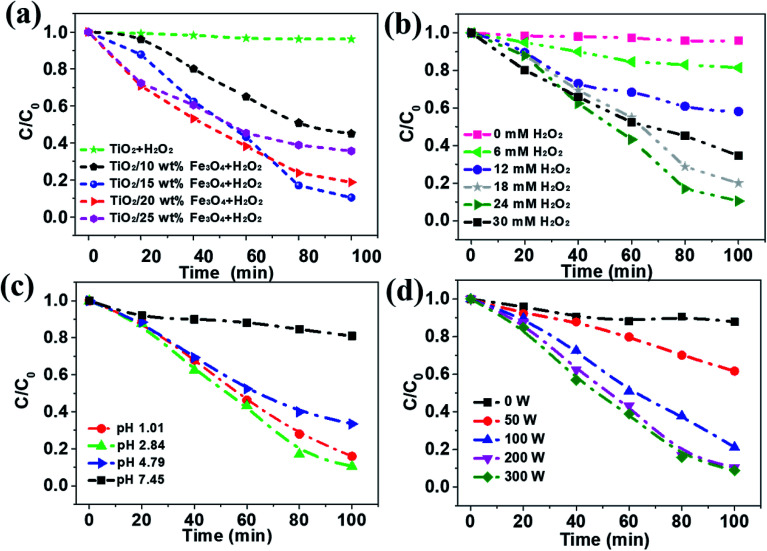
The effect of Fe_3_O_4_ loading (a), H_2_O_2_ concentration (b), different initial pH values (c) and light intensity (d) on the degradation of AMX. Reaction conditions: [AMX] = 30 mg L^−1^ (for a–d), UV irradiation: 200 W (for a, b and c), [H_2_O_2_] = 24 mM (for a, c and d), [TiO_2_/15 wt% Fe_3_O_4_] = 0.4 g L^−1^ (for b, c and d), and pH = 2.84 (for a, b and d).

The removal efficiency of AMX under different conditions was investigated and the results were depicted in Fig. 6. As can be seen from the results, only 3.9% of the organic matter was removed even after 100 min in the absence the activation of Fe_3_O_4_, while the removal efficiency of AMX can increase to 85.0% with 15 wt% Fe_3_O_4_ dosage (Fig. 6a). Then it slightly decreases to 80.0 and 60.5% as the Fe_3_O_4_ content increases to 20% and 25%, respectively. According to Table 1, the BET surface areas of catalysts decrease from 59.5 to 24.8 m^2^ g^−1^ as the loading amount of Fe_3_O_4_ increases from 0 to 25%, which is almost independent of the removal efficiency of AMX. TiO_2_/15 wt% Fe_3_O_4_ showed the highest photo-Fenton catalytic performance. It was attributed to the fact that more Fe^2+^ sites were provided for H_2_O_2_ and thus more active ·OH was generated with the increase of the Fe_3_O_4_ content. However, overloading of Fe_3_O_4_ (TiO_2_/20 wt% Fe_3_O_4_ and TiO_2_/25 wt% Fe_3_O_4_) could block the light absorbance for TiO_2_ photocatalyst. In addition, excessive Fe^2+^ from Fe_3_O_4_ can consume ·OH on occasion of further increase in Fe_3_O_4_ content.^29^ Apparently, almost no obvious decrease of AMX was observed if there was no H_2_O_2_, which is essential in the oxidation of organics in photo-Fenton process (Fig. 6b). Within the first 30 minutes, more H_2_O_2_ corresponded to higher degradation rates. After 30 minutes, it showed the same trend when the concentration of H_2_O_2_ was below 24 mM. Undoubtedly, the increase of H_2_O_2_ resulted in the increase of ·OH, and thus the improvement in Fenton oxidation process. However, further increasing in H_2_O_2_ dosage from 24 to 30 mM, resulted in slight decrease in AMX removal indeed. This observation could be ascribed to the mechanism that excessive H_2_O_2_ could act as a self-scavenger for OH, following eqn (1) and (2), which leads to the greatly decrease of ·OH generation.^30^

1 H_2_O_2_ + ·OH → ·OOH + H_2_O

2 ·OOH + ·OH → H_2_O + O_2_

**Table 1:** The BET surface area of TiO_2_–Fe_3_O_4_ composite prepared with different loading amounts of Fe_3_O_4_

Samples	TiO_2_	TiO_2_/10 wt% Fe_3_O_4_	TiO_2_/15 wt% Fe_3_O_4_	TiO_2_/20 wt% Fe_3_O_4_	TiO_2_/25 wt% Fe_3_O_4_
BET (m^2^ g^−1^)	59.5	48.9	36.8	30.5	24.8

The initial pH of solution plays a vital role in chemical reaction by affecting the charge and other physicochemical property of substance in the mixture. The effect of pH on the photo-Fenton degradation of AMX was evaluated at pH values in the range of 1.01–7.45 (Fig. 6c). It is generally recognized that homogeneous Fenton process happens in acidic conditions and it came to the same conclusion in this photo-Fenton system.^30,31^ The AMX removal increased from 19.1% to 66.6% when the initial pH value decreased from 7.45 to 4.79. With further lower pH, more AMX was degraded and reached the highest at pH 2.84. This can be explained by the scramble of Fe^3+^ between OH^−^ and H_2_O_2_. In other words, more Fe^3+^ will interact with OH^−^ rather than H_2_O_2_ when pH is higher, leading to lower oxidation efficiency. Additionally, the LPML as the UV radiation source was used in the photoreactor. The light intensity in the SMSMPR was investigated to evaluate the kinetics of the photo-Fenton reaction *via* the electron hole formation, separation, and recombination rates (Fig. 6d). It was obvious that the concentration of AMX showed only slight decrease in the dark (light intensity = 0 W), indicating the limited adsorption capacity of TiO_2_/15 wt% Fe_3_O_4_ to AMX. At a low light intensity in the reactor (<200 W), the reaction rate increased remarkably with increasing light intensity, as the generation of electrons and holes was the predominant process. With further increase of light intensity (>200 W), the reaction rate increased slightly, owing to the high electron hole recombination. Meanwhile, electrons may have easily transferred from the catalyst to oxygen under the higher irradiation intensity, resulting in the generation of ·O_2_^−^, which is the rate limiting step for larger TiO_2_ particles. Therefore, light intensity of 200 W is the suitable operating condition for the SMSMPR.

**Fig. 7:**
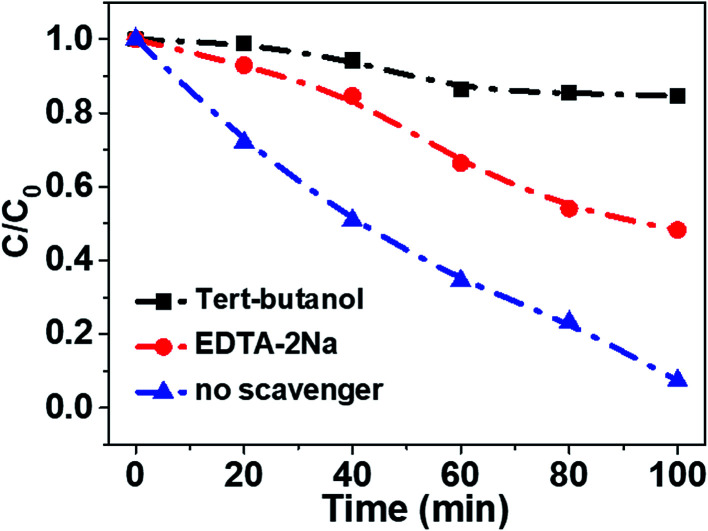
Comparison of photocatalytic activities of TiO_2_/15 wt% Fe_3_O_4_ composite for the TOC removal of AMX with or without adding EDTA-2Na and *tert*-butanol under visible light irradiation. Experimental conditions: [AMX] = 30 mg L^−1^, [H_2_O_2_] = 24 mM, [catalysts] = 0.4 g L^−1^, pH 2.84, and UV light irradiation.

In order to reveal the charge transfer process between TiO_2_ and Fe_3_O_4_, AMX degradation mechanisms by TiO_2_/15 wt% Fe_3_O_4_ composite were further explored. First, the trapping experiments were conducted in the minified system. Specifically, 0.02 g photocatalysts were added into 50 mL AMX solution with a concentration 30 mg L^−1^ in the 100 mL photocatalysis reactor. EDTA-2Na (10 mM) and *tert*-butanol (TBA, 10 mM) were used as a hole scavenger and a hydroxyl radical scavenger (·OH), respectively (Fig. 7). The addition of a scavenger of holes (EDTA-2Na) caused a change in the photodegradation of AMX (50.9%). The degradation of AMX was significantly inhibited in the presence of TBA (15.2%). Thus, it is believed that ·OH and h^+^ should be the main active species in the photo-Fenton degradation of AMX process.

**Fig. 8:**
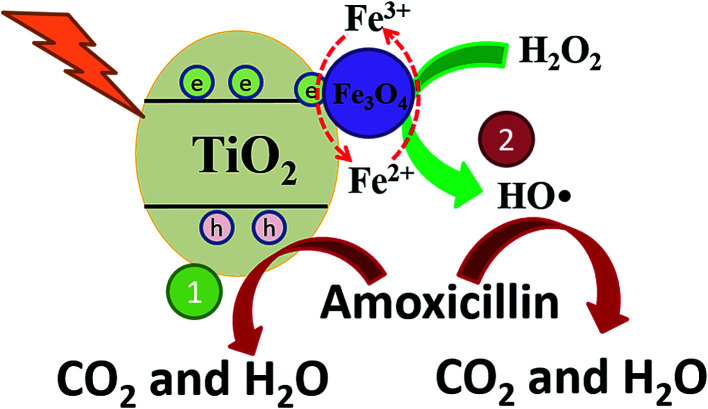
The proposed mechanism of photo-Fenton oxidation of AMX upon TiO_2_/15 wt% Fe_3_O_4_ composite photocatalyst under UV light irradiation.

The mechanism of high AMX degradation in this photo-Fenton system was revealed in Fig. 8. In the TiO_2_/15 wt% Fe_3_O_4_ composite, excited electrons in the TiO_2_ rapidly transfer to the Fe_3_O_4_. The quick separation of photogenerated electron–hole pairs can effectively reduce the h^+^/e^−^ pairs recombination, which contributes to AMX degradation by the h^+^ leaving in the valence band (route 1, Fig. 8). Meanwhile, introduction of Fe_3_O_4_ provided an additional ·OH generation pathway for AMX degradation (route 2, Fig. 8). The redox potential of Fe(ii)/Fe(iii) in Fe_3_O_4_ cycle is effective to activate H_2_O_2_ for the generation of ·OH. More importantly, the photogenerated electron trapped by Fe_3_O_4_ could facilitate the reduction process of Fe(iii) to Fe(ii) and then enhance the Fe(iii)/Fe(ii) cycle during the Fenton process. Therefore, the easier Fe(iii)/Fe(ii) cycle in TiO_2_/15 wt% Fe_3_O_4_ composite contributed to the higher activity and stability for AMX degradation.

**Fig. 9:**
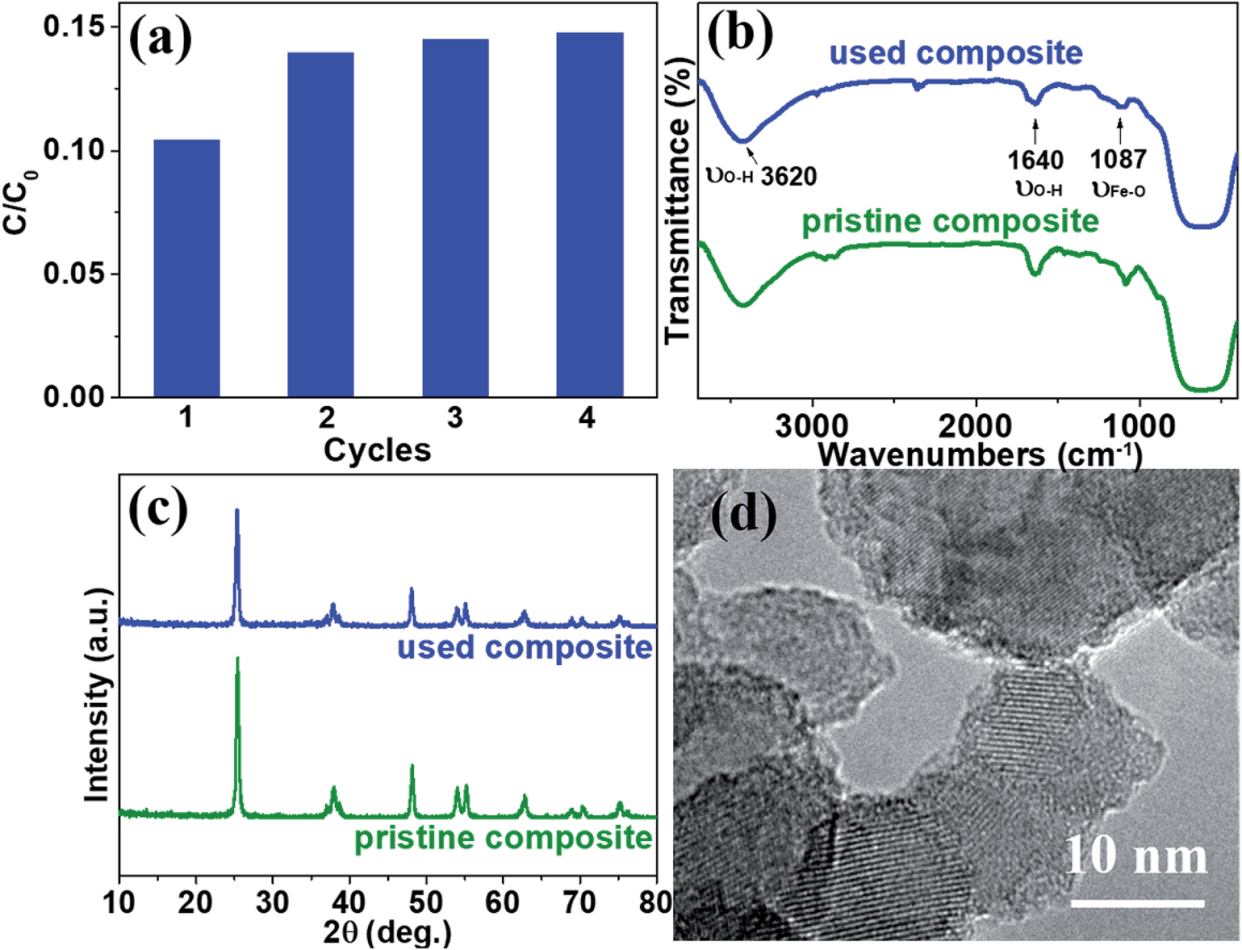
The photo-Fenton degradation of AMX with TiO_2_/15 wt% Fe_3_O_4_ after 4 cycling runs (a), FTIR and XRD of pristine and used TiO_2_/15 wt% Fe_3_O_4_ four times (b and c), and HRTEM of used TiO_2_/15 wt% Fe_3_O_4_ (d). Reaction conditions: [AMX] = 30 mg L^−1^, UV irradiation: 5 mW cm^−2^, [H_2_O_2_] = 24 mM, [TiO_2_/15 wt% Fe_3_O_4_] = 0.4 g L^−1^, and pH = 2.84.

To examine the stability and repeatability of catalyst and SMSMPR, cycle experiments were carried out. After each cycle, an external magnetic field was imposed to the reactor and the photocatalyst was reclaimed, washed and used in next cycle. Fig. 9a presented the AMX degradation rates of four cycles, with the reaction time 100 min for each cycle. Except the first run (maybe because some loosely-combined catalyst leached), there was not significantly declined degradation ability of catalytic composites with more runs and the removal efficiency of AMX was as high as 85.3% even after 4 cycles. We also used FTIR, XRD and HRTEM to evaluate the difference between pristine and used TiO_2_/15 wt% Fe_3_O_4_. There was no obvious difference and the characteristic peaks of OH and Fe–O can be seen in both of the FTIR spectra (Fig. 9b). Fig. 9c illustrated that TiO_2_/15 wt% Fe_3_O_4_ sample after 4 reaction cycles maintained morphology, lattice parameters and crystallinity similar to those of fresh samples. There is no obvious morphology change from HRTEM images (Fig. 9d). These results reveal the high material stability of TiO_2_/15 wt% Fe_3_O_4_ and can be used as an environmentally friendly catalyst for photo-Fenton reaction.

**Fig. 10:**
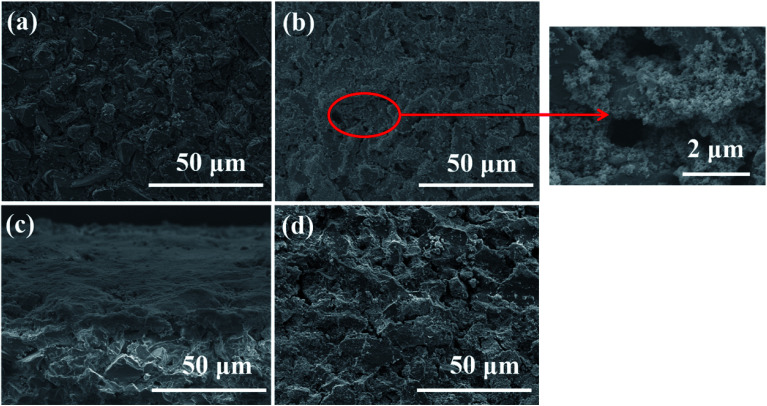
SEM images of the pristine membrane (a), the membrane with deposited catalysts (upper side) (b), the membrane with deposited catalysts (cross-sectional) (c), and the membrane after backwashing treatment and exposure to the external magnetic field (d).

Subsequently, SEM was utilized to investigate the surface change of membrane in the SMSMPR and the results were delineated in Fig. 10. As can be seen, the raw ceramic membrane had rough surface and many micron-scale pores on the surface. After one cycle experiment, the TiO_2_–Fe_3_O_4_ particles were thickly deposited on the surface and even inside the pores of the membrane. However, most of the nanoparticles went away and the membrane recovered clean after backwashing treatment and exposure to the external magnetic field.

It is reported that the deposition of solid and pollutant can increase the trans-membrane pressure, lower the water flux and raise the cost of membrane technology.^32^ We also examined the trans-membrane pressure change during the cycle experiments and the result is given in Table 2. The initial trans-membrane pressure was measured after the cycle experiment started for 5 min and the final trans-membrane pressure was measured after the cycle experiment started for 100 min. As can be observed, the trans-membrane pressure was lifted much higher after one single cycle run. For example, the initial trans-membrane pressure was only 0.47 kPa, but the final was as high as 0.99 kPa after the second cycle started for 100 min. Although backwashing treatment can recover the trans-membrane pressure to some extent, the recovery effect was quite limited. By comparison, the use of external magnetic field maintained the trans-membrane pressure within a relatively low level even after 4 cycles.

**Table 2:** The trans-membrane pressure of each cycle

Pressure (kPa)	Cycle 1	Cycle 2	Cycle 3	Cycle 4
Initial trans-membrane pressure with backwashing only	0.32	0.47	0.55	0.61
Final trans-membrane pressure with backwashing only	0.68	0.99	1.12	1.28
Initial trans-membrane pressure with backwashing and external magnetic field	0.32	0.39	0.41	0.40
Final trans-membrane pressure with backwashing and external magnetic field	0.50	0.51	0.60	0.55

Recently, researchers have used different kinds of magnetic photocatalysts to degrade organic pollutants. Chang *et al.* prepared Fe_3_O_4_/TiO_2_ magnetic photocatalyst for degradation of phenol.^33^ However, they utilized photocatalysis only instead of photo-Fenton process and then the degradation efficiency of organics reached maximum with the catalyst amount as much as 3 g L^−1^. As described above, photo-Fenton system has relatively higher degradation rate of pollutants under acidic conditions in most of the literature and we got the same result in the paper. Actually, researchers have been trying to overcome this drawback by adding chelating agents, such as ethylenediamine-*N*,*N*′-disuccinic acid (EDDS), ethylenediaminetetraacetic acid (EDTA) and nitrilotriacetic acid (NTA) in recent years,^34^ which could be adopted by the system in the paper. Another study also carried out recycling experiments to measure the repetitive use of the catalyst. With even better performance in reusability, the as-prepared Fe_3_O_4_@TiO_2_/SiO_2_ photocatalyst showed just a slight drop (from 94.5% to 90.1%) in removal efficiency after six cycles.^19^ This means that the repeatability of the SMSMPR system may be improved if stronger interaction between Fe_3_O_4_ and TiO_2_ was formed and further study is needed.

## 4. Conclusions

In summary, a novel photocatalysis reactor was successfully built and employed in the photo-Fenton process. Fe_3_O_4_ grown on a TiO_2_–Fe_3_O_4_ composite not only enhances the heterogeneous Fenton degradation of refractory organic compounds but also provides magnetism of the photocatalyst for magnetic separation from treated water. We combined an SMSMPR with the magnetic TiO_2_–Fe_3_O_4_ catalyst. The prepared TiO_2_–Fe_3_O_4_ composites showed high photo-Fenton catalytic activity for degradation of AMX. Cycle experiments demonstrate that the combination of backwashing treatment with magnetic separation could enhance the stability and reusability of the SMSMPR, promoting its practical application for removal of organic pollutants in aqueous solution.

## Conflicts of interest

There are no conflicts to declare.

## Supplementary Material
